# Activation of MITF by Argan Oil Leads to the Inhibition of the Tyrosinase and Dopachrome Tautomerase Expressions in B16 Murine Melanoma Cells

**DOI:** 10.1155/2013/340107

**Published:** 2013-07-08

**Authors:** Myra O. Villareal, Sayuri Kume, Thouria Bourhim, Fatima Zahra Bakhtaoui, Kenichi Kashiwagi, Junkyu Han, Chemseddoha Gadhi, Hiroko Isoda

**Affiliations:** ^1^Alliance for Research on North Africa (ARENA), University of Tsukuba, Tennodai 1-1-1, Tsukuba City, Ibaraki 305-8587, Japan; ^2^Graduate School of Life and Environmental Sciences, University of Tsukuba, Tennodai 1-1-1, Tsukuba City, Ibaraki 305-8587, Japan; ^3^Faculty of Sciences Semlalia, Cadi Ayyad University, Avenue Prince Moulay Abdellah, BP 2390, 40000 Marrakesh, Morocco

## Abstract

Argan (*Argania spinosa* L.) oil has been used for centuries in Morocco as cosmetic oil to maintain a fair complexion and to cure skin pimples and chicken pox pustules scars. Although it is popular, the scientific basis for its effect on the skin has not yet been established. Here, the melanogenesis regulatory effect of argan oil was evaluated using B16 murine melanoma cells. Results of melanin assay using B16 cells treated with different concentrations of argan oil showed a dose-dependent decrease in melanin content. Western blot results showed that the expression levels of tyrosinase (TYR), tyrosinase-related protein 1 (TRP1), and dopachrome tautomerase (DCT) proteins were decreased. In addition, there was an increase in the activation of MITF and ERK1/2. Real-time PCR results revealed a downregulation of *Tyr*, *Trp1*, *Dct*, and *Mitf* mRNA expressions. Argan oil treatment causes MITF phosphorylation which subsequently inhibited the transcription of melanogenic enzymes, TYR and DCT. The inhibitory effect of argan oil on melanin biosynthesis may be attributed to tocopherols as well as the synergistic effect of its components. The results of this study provide the scientific basis for the traditionally established benefits of argan oil and present its therapeutic potential against hyperpigmentation disorders.

## 1. Introduction 

 The use of argan oil to moisturize the skin and to maintain a fair complexion has been an established tradition among Moroccan women.**** *Argania spinosa* (L.) Skeels (Sapotaceae) is a tree species endemic to Morocco and has a great ecological and socioeconomic value in this area. The fruit of *A. spinosa* has an oleaginous kernel from which a well-known oil, “argan oil,” is used in folk medicine and in cosmetics [1], especially in the southwestern region [2]. Traditionally, cosmetic argan oil was used to cure all kinds of skin pimples as well as juvenile acne and chicken pox pustules scars. It is also recommended to reduce dry skin matters and slow down the appearance of wrinkles. This oil is also used to treat psoriasis, eczema, joint pain, skin inflammation, and scabies, to heal burns and wounds, to cure brittle fingernails, to prevent hair loss and dry hair [3]. Argan oil for cosmetic use is cold-pressed oil extracted from unroasted kernels of argan fruit, which at present has gained worldwide recognition. It has been referred to as the “most expensive vegetable and cosmetic oil” [4] and as such has been a source of income for many Moroccan women who are members of cooperatives run by women. In the southern part of Morocco, cooperatives produce argan oil for food or for cosmetic use. The extraction of argan oil for food and cosmetic use is still being done using the traditional method. Argan oil is rich in tocopherols, medium chain fatty acids, carotenoids [5–7], squalene, and oleic acid [8]. 

 Acquired hyperpigmentation after exposure to sunlight for a long time or such as in melasma and postinflammatory melanoderma is characterized by an increase in production and accumulation of melanin [9]. The biosynthesis of melanin in the pigment cells is catalyzed by the melanogenic enzymes tyrosinase, tyrosinase-related protein 1, and dopachrome tautomerase [10, 11]. Hyperpigmentation occurs when there is an increase in the activity of these enzymes due to triggering factors such as ultraviolet light or chronic inflammation [12, 13]. Different approaches to address this problem have been the subject of most researches [14–17]. Depigmenting agents are applied not just for the prevention and treatment of irregular hyperpigmentation in Western countries, but to make the skin whiter based on traditional beliefs in Asia [18]. 

 The use of chemical agents such as hydroquinone and kojic acid, among many others, is the most popular way of managing hyperpigmentation [9, 19, 20]. However, undesirable side effects such as allergy [21], exogenous ochronosis, infectious dermatosis, and, in particular, extended dermatophytosis and necrotizing cellulitis, contact eczema, and certain hyperpigmentation when depigmenting agents were discontinued [22]. It is for these unwanted effects that the use of naturally occurring and locally available depigmenting agents still seems to be the most practical way to manage hyperpigmentation [23]. 

 The depigmenting effect of the components of argan oil such as free fatty acids, tocopherols [24, 25], and the ability of carotenoids to protect the skin from sunlight [26] has been reported. However, there has been no study on the effect of whole argan oil on the melanin biosynthesis using a murine cell culture model. This study presents the use of argan oil as a depigmenting agent and the elucidation of the mechanism underlying its effects is also presented. 

## 2. Materials and Methods 

### 2.1. Origin of Argan Oil Sample and Oil Components Composition Analysis

Argan oil used in the experiment was kindly provided by Alain-Claude Kerrien (Naturelle d'argan, Marrakech, Morocco). It was cosmetic-type argan oil obtained, in a women argan cooperative (Cooperative Agdal, Essaouira Region, Morocco), by mechanical press of unroasted almonds of argan tree (*Argania spinosa L. Skeels, *Sapotaceae). 

 Its fatty acid and minor components composition were determined according to standard procedures: NF EN ISO 5508 for fatty acids composition (methyl esters in %), ISO 9936 for the composition of tocopherols, and NF ISO 6799 for the composition of sterol fraction. 

### 2.2. Antioxidant Activity of Argan Oil

#### 2.2.1. *β*-Carotene/Linoleic Acid Bleaching Assay

 The *β*-carotene/linoleic acid bleaching assay was performed following the method described by Miraliakbari and Shahidi [27] with slight modification. A mixture of *β*-carotene and linoleic acid was prepared by dissolving 0.5 mg *β*-carotene in 1 mL chloroform (HPLC grade) and 25 *µ*L linoleic acid in 200 mg Tween 20. The chloroform was then completely evaporated under vacuum, and 100 mL of distilled water was subsequently added to the residue, and then the mixture was shaken vigorously to form an emulsion. From this emulsion, 2.5 mL was transferred into different test tubes containing 350 *µ*L of samples in acetone at different concentrations. All samples were vortexed for 1 min and placed at 50°C in a water bath for 2 h together with a negative control (blank) which contained the same volume of acetone instead of the samples. The absorbance of samples was measured at 470 nm using a spectrophotometer at initial time (*t* = 0) against a blank (emulsion without *β*-carotene). A standard BHT was used as a control.

Antioxidant activities (inhibitions percentage, I%) of the samples were calculated using the following equation:

(1) $$\begin{array}{c} \text{I}\%=\left(\frac{{\text{A}}_{\beta \text{-carotene}\;\text{after}\;2\;\text{h}\;\text{assay}}}{{\text{A}}_{\text{initial}\;\text{of}\;\beta \text{-carotene}\times 100}}\right)\times 100,\;\; \end{array}$$

where A_*β*-carotene  after  2 h  assay_ is the absorbance values of *β*-carotene after 2 h assay remaining in the samples and A_initial  of  *β*-carotene_ is the absorbance value of *β*-carotene at the beginning of the experiments.

All tests were carried out in triplicate, and inhibition percentages were reported as means SD of triplicates.

#### 2.2.2. Reducing Power Activity

 The reductive potential of argan oil was determined following the method of Bounatirou et al. [28]. Different concentrations of argan oil in acetone (1 mL) were mixed with phosphate buffer (2.5 mL, 0.2 M, and pH 6.6) and potassium ferricyanide (2.5 mL, 1% w/v). The mixture was incubated at 50°C for 20 min. Trichloroacetic acid (10%, 2.5 mL) was added to the mixture and centrifuged for 10 min at 1200 g. The upper layer of the solution (2.5 mL) was mixed with distilled water (2.5 mL) and FeCl_3_ (0.5 mL, 0.1%), and the absorbance was measured using a spectrophotometer (700 nm). The extract concentration providing 0.5 of absorbance (IC50) was calculated by plotting the absorbance at 700 nm against the corresponding argan concentration. BHT and ascorbic acid were used as reference compounds.

### 2.3. Cell Culture

 The B16 murine melanoma cells (B16 cells) used in the experiment were purchased from the Riken Cell Bank in Tsukuba, Japan, and maintained as a monolayer culture in Dulbecco's Modified Eagle's Medium (Nissui, Tokyo, Japan) supplemented with 10% fetal bovine serum (Sigma, St. Louis, MO, USA), 4 mm l-glutamine (Sigma), 50 units/mL penicillin, and 50 *μ*g/mL streptomycin (Cambrex, East Rutherford, NJ, USA) at 37°C in a humidified atmosphere of 5% CO_2_.

### 2.4. Melanin Quantification

 The melanin content synthesized by B16 melanoma cells treated with argan oil was determined as previously described [16]. B16 cells were seeded onto 100 mm petri dishes at a density of 5 × 10^5^ cells/dish and incubated at 37°C in a 5% CO_2_ atmosphere. After overnight incubation, the growth medium was replaced with a fresh medium containing arbutin (100 *µ*M) or argan oil (1/100, 1/1,000, or 10,000 v/v) dilutions. After 48 h incubation, the growth medium was then removed, and then the cells washed twice with phosphate-buffered saline (PBS) and harvested by trypsinization (0.25% trypsin/0.02% EDTA in PBS; Sigma). The cells were pelleted and 0.1% Triton X-100 was added to solubilize the cell membrane. The synthesized melanin was then purified and precipitated in 10% trichloroacetate, and the melanin dissolved in 1 mL 8 N NaOH for 2 h at 80°C. The absorbance of the melanin solution was measured at 410 nm, and the melanin content was calculated using a standard curve for synthetic melanin. The cell viability and total cell count were assessed using the ViaCount Program of Guava PCA (GE Healthcare, UK Ltd., Buckinghamshire, UK) following the manufacturer's instructions. The melanin content was expressed as melanin content/cell (% of control). 

### 2.5. Western Blot

The protein samples used for western blotting were extracted from B16 cells seeded onto 100 mm dishes at a density of 3 × 10^6^ cells per dish and cultivated as described above. After the cells were allowed to attach overnight, the medium was replaced with medium containing hirsein A (HA), a melanogenesis inhibitor [17], or argan oil (1/10,000, 1/1,000, or 1/100) and incubated for additional 24 or 48 h. The medium was then removed, and the cells washed twice with PBS before the total protein was extracted using RIPA buffer (Sigma, USA) according to the manufacturer's instructions. Protein sample (15 *μ*g) was resolved in 10% SDS-polyacrylamide gel by polyacrylamide gel electrophoresis, transferred to nitrocellulose membrane, and blotted with primary antibodies for tyrosinase, (MITF) microphthalmia-associated transcription factor, tyrosinase (TYR), tyrosinase-related protein 1 (TRP1), dopachrome tautomerase (DCT), p-ERK1/2 (Sigma), ERK2 (Sigma), and GAPDH (Santa Cruz Biotechnology, Inc., USA). The signal was visualized using LiCor Odyssey Infrared Imaging System after reaction with goat anti-mouse IRDye 680LT, donkey anti-goat IRDye 800CW (LI-COR), or goat anti-rabbit IRDye 800CW (LI-COR).

### 2.6. Quantitative Real-Time PCR Analysis

B16 cells were seeded onto 100 mm dishes at a density of 3 × 10^6^ cells per dish and cultivated as described above. After the cells were allowed to attach overnight, the medium was then replaced with medium with or without argan oil (1/10,000, 1/1,000, or 1/100) and incubated for additional 24 h. The medium was then removed, and the cells washed twice with PBS before the total RNA was extracted using ISOGEN kit (Nippon Gene, Tokyo, Japan) and quantified using a NanoDrop 2000 spectrophotometer (NanoDrop Technologies). The RNAs were then used as templates for reverse transcription polymerase chain reaction (RT-PCR) using the SuperScript III reverse transcriptase kit (Invitrogen, Carlsbad, CA, USA) following the manufacturer's instructions. To quantify the mRNA expression of the tyrosinase (*Tyr*) gene, quantitative real-time polymerase chain reaction (RT-PCR) analysis was performed with a 7500 Fast Real-Time PCR system using TaqMan Universal PCR mix and TaqMan probes (Applied Biosystems, Foster City, CA, USA) with the following thermal cycling protocol: 95°C for 10 min followed by 40 cycles of 95°C for 15 s and 60°C for 1 min. *Gapdh *was used as a control. Specific TaqMan primers for *Tyr, Trp1, Dct, Mitf*, and *Gapdh* were obtained from Applied Biosystems (Foster City, CA, USA). Data were analyzed using 7500 Fast System SDS Software 1.3.1. (Applied Biosystems). 

### 2.7. Statistical Analysis

Results were expressed as mean value ± SD, and the differences between treated and control treatments were evaluated for significance using Student's *t*-test. Differences between the means at the 5% confidence level were considered significant.

## 3. Results

### 3.1. Chemical Analyses

 The chemical analyses of the cosmetic argan oil used in the experiments were performed.  Table 1 shows the amount of the major fatty acid in argan oil oleic acid (46.1%), linoleic acid (34.5%), palmitic (12.2%), and stearic acid (6.23%). The amount of tocopherols present in the sample was also evaluated and results showed that the total tocopherol was 63.5 mg/100 g oil with alpha-tocopherol as the main tocopherol (44 mg/100 g oil) (Table 2). The chemical analysis also revealed the presence of triterpene alcohols, carotenoids and xanthophylls. The sterols present in argan oil are *β*-sitosterol (highest concentration), spinasterol, schottenol, and *β*-sitosterol. Other components include carotenoids (2 mg/kg) and xanthophylls (1.7 mg/kg)**  **(data not shown). 

### 3.2. Antioxidant Activity and Reductive Potential of Argan Oil

 The antioxidant activity of argan oil was determined using the *β*-carotene–linoleate model system and compared with that of BHT. The results showed that argan oil prevented the bleaching of *β*-carotene and therefore may contribute to the lipoperoxidation protection (Table 3). Furthermore, to determine argan oil's ability to act as an electron donor, its reductive potential was also evaluated. The electron donor reacts with free radicals, converts them to more stable products, and finally terminates radical chain reactions. The reductive capacity of a compound is recognized as a significant indicator of its potential antioxidant activity [28]. Argan oil showed some degree of electron-donating capacity, but it was still lower than that of synthetic antioxidant BHT (EC50 = 31.33 ± 0.03 *µ*g/mL) and ascorbic acid (EC_50_ = 6.89 ± 0.01 *µ*g/mL). 

### 3.3. Effect on the Melanin Content in B16 Cells

**Figure 1:**
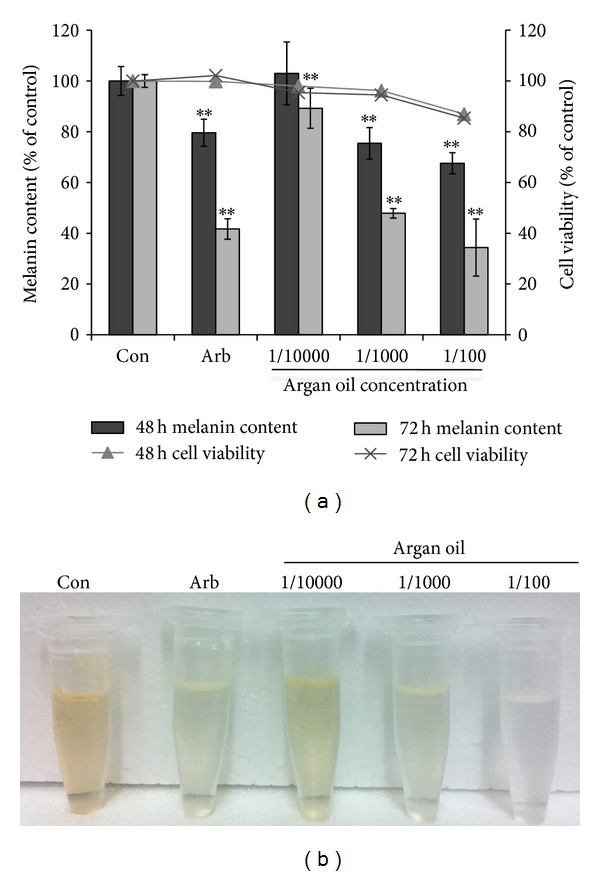
Effect of argan oil (AO) at 1/10000, 1/1000, and 1/100 (v/v) on the (a) melanin content (bar graph) and cell viability (line graph) of B16 melanoma cells cultured in 100 mm dish at a density of 5 × 10^5^ cells/dish. (b) Lysates of cells treated with or without arbutin (100 mM) or argan oil. B16 cells were treated with or without arbutin or argan oil serially diluted in Dulbecco's Modified Eagles Medium and incubated for 48 or 72 h prior to melanin assay. *Statistically significant (*P* < 0.05) difference between treated and untreated (Con) cells.

 The amount of intracellular melanin in B16 cells treated with argan oil was quantified. Argan oil was mixed with the growth medium and sonicated prior to treatment. Cells treated with 1/100, 1/1000, and 1/10000 v/v of argan oil were then incubated for 48 h and the melanin content was quantified as described in the Materials and Methods Section. Results showed that treatment with argan oil caused a dose-dependent decrease in the melanin content of B16 cells. Cell viability assay results revealed that except for 1/100 dilution, argan oil does not have any cytotoxic effect on B16 cells. Longer incubation time (72 hours) revealed a time-dependent significant decrease in the melanin content of the cells (Figure 1). Compared to arbutin, a known melanogenesis inhibitor, cells treated with argan oil for 72 h had almost the same amount of melanin as the arbutin-treated cells. The color of the cell lysates dissolved in NaOH clearly shows the depigmenting effect of argan oil (Figure 1(b)).

### 3.4. Melanogenic Enzymes Expressions in B16 Cells

**Figure 2:**
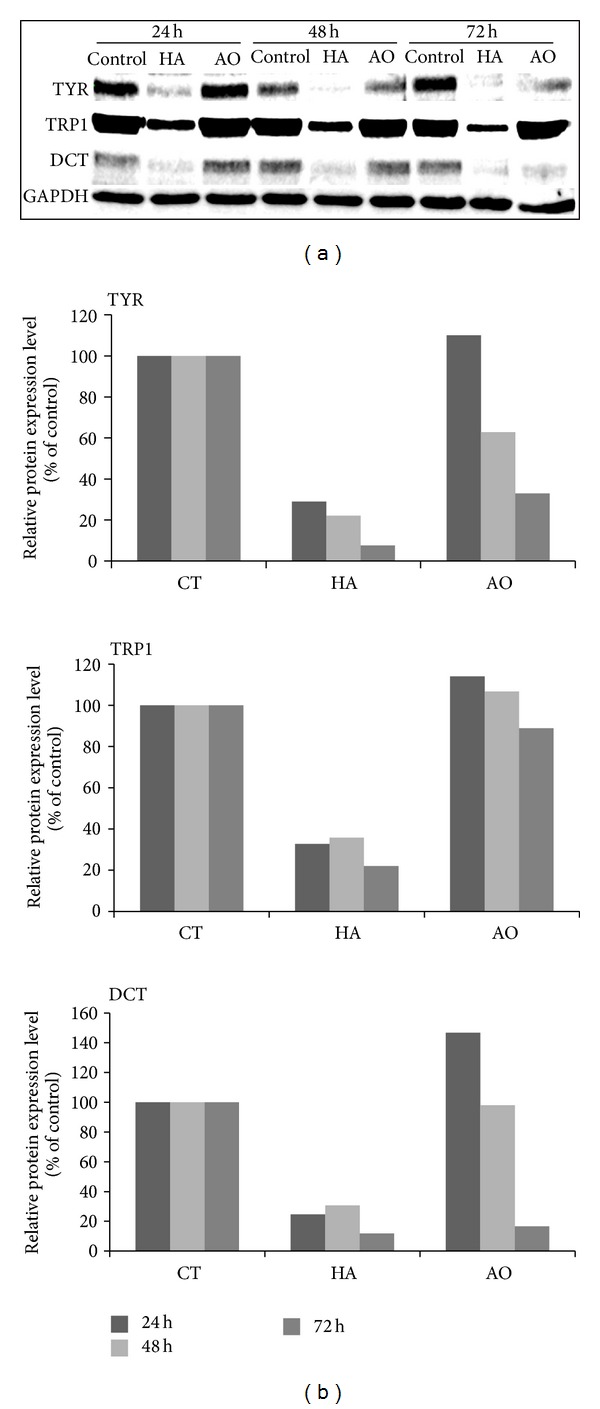
Effect of argan oil on the expressions of the tyrosinase (TYR), tyrosinase-related protein 1 (TRP1), and dopachrome tautomerase (DCT) proteins determined by western blotting (a) and their densitometric values (b). B16 melanoma cells were cultured in 100 mm dish at a density of 3 × 10^6^ cells/dish and treated without (Con or CT) or with 1 *µ*M hirsein A (HA) or 1/1000 v/v argan oil (AO).

 Melanin biosynthesis is catalyzed by three major enzymes: tyrosinase (TYR), tyrosinase-related protein 1 (TRP1), and dopachrome tautomerase (DCT). Here, the expressions of these enzymes were determined by western blotting using specific antibodies, and the results showed that treatment with 1/1000 v/v argan oil inhibited the expression of tyrosinase and Dct proteins in a time-dependent manner, the significant inhibition of which was at after 72 hrs of treatment (Figure 2(a)). Like tyrosinase, there was a significant decrease in DCT expression which was not observed in TRP1. Densitometric analysis of the protein expressions showed that there was about 60% decrease in TYR expression and 80% in DCT protein levels (Figure 2(b)). It was, however, noted that the expression of TRP1 was not significantly decreased with only about 10% decrease in its expression after 72 h of treatment with argan oil (compared to the untreated control). As expected, the positive control hirsein A significantly inhibited the expressions of all the three enzymes [17]. 

### 3.5. Molecular Mechanism Underlying the Depigmenting Effect

**Figure 3:**
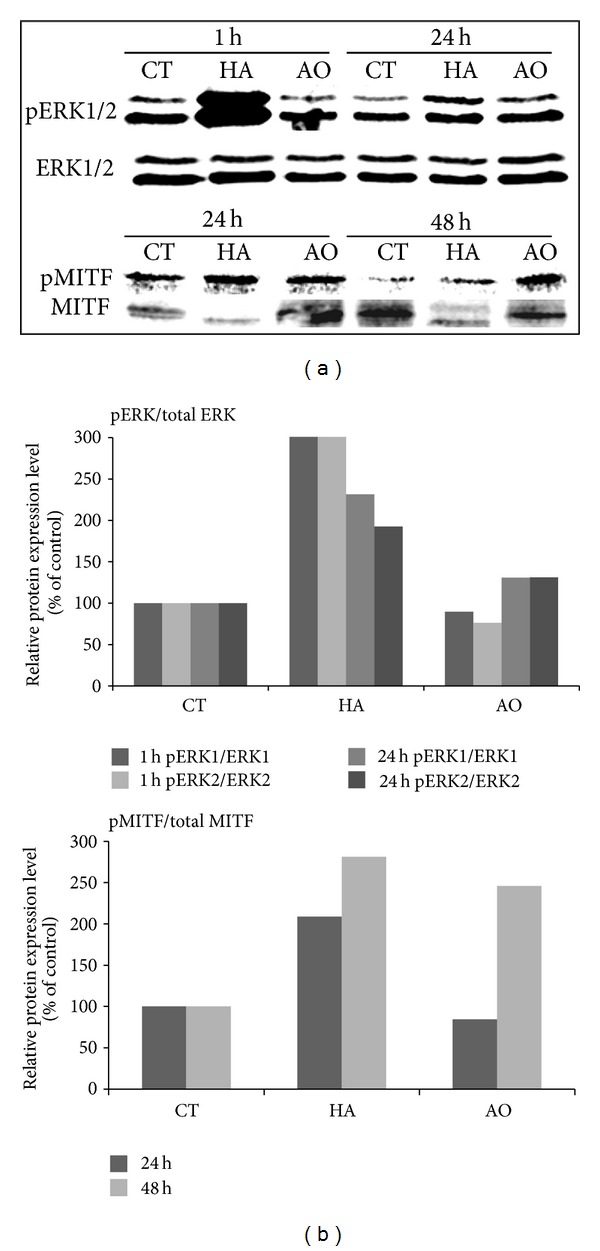
Effect of argan oil on the expressions of the phosphorylated extracellular signal-regulated kinases 1/2 (pERK1/2), ERK1, ERK2, phosphorylated microphthalmia-associated transcription factor (pMITF), and total MITF proteins (a) and their densitometric values (b). B16 melanoma cells were cultured in 100 mm dish at a density of 3 × 10^6^ cells/dish and treated without (CT) or with 1 *µ*M hirsein A (HA) or 1/1000 v/v argan oil (AO).

 The expressions of the melanogenic enzymes' genes are regulated by the MITF that binds on the regulatory element of *Tyr*, *Trp1*, and *Dct* genes. The expressions of total and phosphorylated ERK1/2 and MITF were evaluated using western blot, the results of which revealed a significant increase in the activation of ERK 24 h after argan oil treatment (Figure 3(a)). Compared to hirsein A which had significant ERK phosphorylation 1 h after treatment, argan oil appears to induce this phosphorylation after 24 h. Likewise, an increase in the level of phosphorylated MITF expression (Figure 3(a)) and the ratio of the phosphorylated to the total MITF was observed 48 h after treatment with argan oil (Figure 3(b)). 

**Figure 4:**
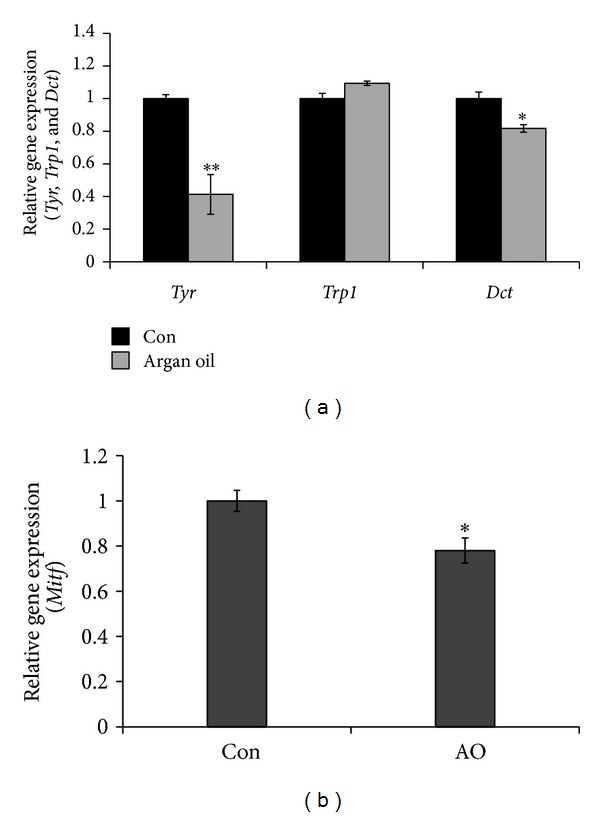
Effect of argan oil on the (a) tyrosinase (*Tyr*), tyrosinase-related protein 1 (*Trp1*), and dopachrome (*Dct*) and (b) microphthalmia-associated transcription factor (*Mitf*) mRNA expressions quantified using TaqMan real-time PCR. B16 melanoma cells were cultured in 100 mm dish at a density of 3 × 10^6^ cells/dish and treated without (Con) or with 1/1000 v/v argan oil and incubated for 24 h prior to total RNA extraction.

 To confirm if the increase in the phosphorylation of MITF led to a decrease in the expressions of the *Tyr*,* Trp1*, or* Dct* mRNA, real-time PCR was performed. Results showed that argan oil significantly downregulated the expressions of *Tyr* and *Dct* mRNA but not *Trp1* (Figure 4(a)). To determine if argan oil has an effect on the expression of MITF at the transcriptional level, the expression of *Mitf* gene was quantified using real-time PCR. Results showed a downregulation of the *Mitf * mRNA expression (Figure 4(b)).

## 4. Discussion

 The health benefits of argan oil consumption have been widely reported. Argan oil confers cancer chemopreventive [8] and hypolipidemic effects [29], among others. Here, the effect of argan oil on the skin was tested *in vitro* using the model cell line B16 murine melanoma cells. Argan oil has been used by Moroccan women as cosmetic oil for centuries, but it is only until recently that the demand for argan oil has spread beyond North Africa. European chemists recently discovered that argan oil exhibits desirable biochemical properties for cosmetics sold commercially throughout Europe, Israel, and around the world [30]. 

 Skin pigmentation depends on the amount of melanin and its deposition on the skin. During melanin production, pigment cells, such as B16 cells, depend on the amount of tyrosinase in the cells or on compounds/factors that inhibit or activate this enzyme [31–33]. Arbutin, kojic acid, and ascorbic acid are just a few of known compounds that can inhibit melanin biosynthesis [34]. The effectivity of depigmenting agents on melanogenesis is evaluated by quantifying the melanin content of the pigment cells. The biosynthesis of melanin occurs in the melanosomes that contain the enzymes tyrosinase (TYR), tyrosinase-related protein 1 (TRP1), and dopachrome tautomerase (DCT). The first two rate-limiting reactions of melanogenesis are catalyzed by tyrosinase and these are (1) the hydroxylation of tyrosine giving the 3, 4-dihydroxyphenylalanine (DOPA) and (2) the oxidation of dopa to DOPAquinone. In mice, Trp1 has been shown to oxidize 5, 6-dihydroxyindole-2-carboxylic acid (DHICA) to indole-5, 6-quinone-2-carboxylic acid, which is not observed in human TRP1. DCT isomerizes dopachrome to DHICA [34].

 In this study, we evaluated the effect of argan oil on melanogenesis to validate the traditional belief that it can maintain a fair complexion. Argan oil, mixed with the growth medium of B16 cells incubated for 48 to 72 hours, decreased the melanin content of the cells (Figures 1(a) and 1(b)). The observed depigmentation was found to be due to the decrease in the levels of the melanogenic enzymes, with a significant decrease in TYR and DCT than in TRP1 (Figure 2). Tyrosinase is the rate-limiting enzyme of melanogenesis and is transported to the melanosomes where it mediates the first two steps of melanin synthesis. The decrease in the melanin content was observed after 48 h of treatment, but the most significant inhibitory effect was observed after 72 h. Although 1/10000 dilution can already cause a depigmenting effect, significant melanin synthesis inhibitory effect without cytotoxicity was observed in 1/1000 dilution, even when compared to 100 *µ*M arbutin, a known melanogenesis inhibitor [35]. The effect of argan oil at 1/100 dilution was also significant but also caused a slight decrease in the cell count and cell viability. We then determined the expression of tyrosinase as affected by argan oil treatment by western blot. Results show that the expression of the tyrosinase enzyme was decreased when cells were treated with argan oil (Figure 2). Although an immediate decrease in the concentration, such as after 4 h of treatment (Figure 2(a)), was not observed prolonged incubation with argan oil caused a significant decrease in the enzymes expressions (Figure 2(b)). Argan oil appears to not have any inhibitory effect on TRP1 and this is not surprising because although TRP1, and DCT share ~40% amino acid homology with TYR*  *[36, 37], *Trp1* has a structure and promoter sequence very different from *Tyr *[38]. However, the reason for the observed effect of argan oil on TRP1 expression remains unclear. 

 In order to understand the underlying mechanism for the decrease in protein levels of the melanogenic enzymes, their expressions at the transcription level were also determined and the results show that their mRNA levels were also decreased by argan oil treatment (Figures 3(a) and 3(b)). The control of the expression of *Tyr*, the rate-limiting melanogenesis, depends in part on the regulation at the transcriptional level [39]. Some melanogenesis inhibitors such as TPA have been found to decrease the *Tyr* mRNA levels in treated cells [40], and in the case of argan oil, the same phenomenon was observed. As shown in Figure 4(a), the level of expression of the *Tyr* mRNA was decreased following argan oil treatment. 

 Melanogenic enzymes' gene expressions are regulated by the MITF, and our results showed an increase in the activation of MITF. The phosphorylation of MITF is linked to its subsequent degradation [41]. MITF plays an important role in melanocyte differentiation, proliferation, and survival [42]. To determine the probable cause of the increase in the activation of MITF, a time-dependent increase in the phosphorylation of ERK was performed the results of which show an increase in ERK1/2 activation by argan oil treatment (Figure 3). Compared to hirsein A, observed to have increased phosphorylation after one hour of treatment, argan oil appears to significantly increase ERK phosphorylation after 24 h of treatment. It has been established that sustained activation of the MAPK ERK leads to MITF degradation [41, 43]. Upon activation, the ERKs either phosphorylate a number of cytoplasmic targets or migrate to the nucleus where they phosphorylate and activate a number of transcription factors such as MITF. MAP kinase, ERK2, phosphorylates MITF, thereby targeting the transcription factor to proteasomes for degradation. Thus, in addition to the complex transcriptional regulation, melanogenesis is also subjected to a posttranslational regulation that controls MITF or TYR function [44]. 

 As summarized in Tables 1, 2, and 3, argan oil is rich in tocopherols and unsaturated fatty acids such as oleic acid and linoleic acid. Argan oil shows an interesting antioxidant activity in *β*-carotene/linoleic acid bleaching assay, predicting a lipoperoxidation protection. The skin whitening effect of alpha-tocopherol or commonly known as vitamin E has been reported [45]. Oral intake of vitamin E can improve facial hyperpigmentation, especially in combination with vitamin C. The exposure to UV radiation induces lipid peroxidation in the skin, which can cause damage to the epidermal cells, leading to postinflammatory hyperpigmentation. This depigmenting effect of alpha-tocopherol is attributed to its antioxidative effects [46] and tyrosinase activity inhibitory effects [47]. 

 The antioxidant and reducing properties of argan oil provide additional benefit to the cells via the reduction of oxidative stress in the absence of melanin. Melanin has a free radical scavenging property in pigment cells. In conditions where melanin is decreased, such as in cells treated with argan oil or in melanocytes deprived of melanin such as in OCA2 cells, oxidative stress inhibitors may provide protection from oxidative damage [48, 49]. Fujiwara et al. [48] reported that vitamin E or tocopherol simultaneously administered with vitamin C traps the peroxyradical caused by UV-B, acts as a radical scavengers and inhibit, the production of melanogenic cytokines, and this appears to be due to the synergistic effect of vitamins A and C on B16 melanoma cells. This mechanism could also explain how, in this study, the different components of argan oil worked collectively to inhibit melanin synthesis in B16 cells. Argan oil, as well as its components, is known to have antioxidant and antiaging properties and can therefore alleviate oxidative stress in the cells, but this effect could be independent of its effect on melanogenesis. Alpha-MSH, for example, a known melanogenesis stimulator, has an oxidative stress inhibitory effect on human melanocytes and even on cells that have lost their ability to produce melanin (cells with *OCA2* mutation) providing evidence that its antioxidant effect may not be directly related to its melanogenesis regulatory effects [49]. The antioxidant effect of argan oil may serve to protect the cells from oxidative damage from UV radiation in the absence of melanin following its depigmenting effect on the skin, thus giving a scientific basis to the traditionally claimed effect on the reduction of the rate of appearance of wrinkles and in fighting dry skin [3].

 Although tocopherols appear to be the main component that influences the overall effect of argan oil on melanogenesis, the effect of other components may also play an important role. Argan oil also contains free fatty acids with reported effects on tyrosinase activity. Linoleic acid has been reported to decrease tyrosinase activity, while palmitic acid increases it [40]. The argan oil sample used in this study has 28.47% linoleic acid, and 12.2% palmitic acid and although it can be induced that the high linoleic content can cause a decrease in the tyrosinase activity, the results of this study showed that argan oil does not affect mushroom tyrosinase activity (data not shown). Obviously, and as reported by Ando et al. [40], tyrosinase activity is not correlated with the *Tyr* mRNA level. The overall skin depigmenting effect due to the inhibition of melanogenic enzymes (TYR and DCT) expressions can therefore be attributed to the main components (tocopherols) and/or the synergistic effect of all the argan oil components. 

## 5. Conclusion

 There is a renewed interest in plants as pharmaceuticals in the Western world and this is on the discovery of new biologically active molecules and into the adoption of crude extracts of plants for self-medication by the general public [47]. North African traditional medicine, therefore, has an important contribution in the maintenance of health in all parts of the world and in the introduction of new treatments. And although some may view the isolation of compounds and their use as single chemical entities as a better option and has resulted to the replacement of plant extracts use, nowadays, a view that there may be some advantages to the medical use of extracts as opposed to isolated single compounds, is gaining popularity [50, 51]. Published reports on the therapeutic benefits of consumption of argan oil have been summarized by [52], but its effect on pigment cells has not yet been reported. Here, we present argan oil as an effective melanin biosynthesis inhibitor, the effect of which may be contributed by the individual components or their synergistic effect. Although the effect of argan oil components (tocopherols, fatty acids, etc.) on melanin biosynthesis has been reported, this is the first report on the inhibitory effect of argan oil on melanogenesis in B16 cells, providing the scientific basis for the centuries-long knowledge of its beneficial effect on the skin. 

## Figures and Tables

**Table 1:** Major fatty acids content of the argan oil used in the experiment.

Fatty acid	Amount (%)
Palmitic acid	12.2
Stearic acid	6.23
Oleic acid	48.5
Linoleic acid	28.47

**Table 2:** Tocopherols present in the argan oil used in the experiment^a^.

Tocopherols	(mg/100 g argan oil)
Total tocopherols	63.5
Alpha-tocopherol	44
Beta-tocopherol	10
Delta-tocopherol	8
Gamma-tocopherol	1

^a^Analysis performed by the Etablissement Autonome de Controle et de Coordination des Exportations (EACC) “Agadir, Morocco.”

**Table 3:** Antioxidant activity of argan oil and synthetic antioxidant (BHT and ascorbic acid) in *β*-carotene bleaching assay and reducing power methods.

Sample	*β*-carotene (EC_50_, *µ*g/mL)	Reducing power (EC_50_, *µ*g/mL)
Argan oil	93.02 ± 5.74	800.31 ± 0.8
BHT	50.31 ± 0.07	31.33 ± 0.03
Ascorbic acid		6.89 ± 0.01
